# Effectiveness and Learner Perception of a Gamified Distance-Learning Program for Point-of-Care Ultrasound

**DOI:** 10.7759/cureus.111035

**Published:** 2026-06-17

**Authors:** Neil Wallace, Bianca K Jao, Veronica Khauv, Arthur B Sanders, Srikar Adhikari, Elaine Situ-LaCasse

**Affiliations:** 1 Emergency Medicine, University of Arizona, Tucson, USA; 2 Pediatrics, University of Arizona, Tucson, USA

**Keywords:** gamification in medical education, medical student training, online medical education, pocus in emergency medicine, point-of-care ultrasound (pocus)

## Abstract

Objectives

The COVID-19 pandemic disrupted in-person medical school teaching, and certain parts of the curriculum were disproportionately affected, including traditional hands-on training in point-of-care ultrasound (POCUS). It is challenging to find interactive methods for teaching POCUS while conveying image acquisition techniques via online video-conference platforms for distance learning. Our objective was to evaluate the effectiveness and learners perceptions of POCUS gamification distance learning.

Methods

This was a cross-sectional study at an academic center. Study participants were third-year medical students (MS3) with minimal ultrasound experience. A pre-test was administered. Point-of-care ultrasound (POCUS) fellowship-trained emergency medicine (EM) faculty gave an online 1-h review lecture on basic POCUS applications and ran an online POCUS Pictionary session, where a student was randomly chosen to illustrate a POCUS topic. After each turn, the instructor reviewed teaching points. Students completed a post-test and survey. Descriptive statistics were used to summarize the data. Survey responses were reported as percentages of total respondents with 95% confidence intervals, and a two-sample t-test was performed to determine the statistical significance of pre- and post-test performances.

Results

A total of 73 students completed the pre-test, post-test, and survey. The average pre-test score was 53.3% (±15.9%), and the post-test score was 84.0% (±13.2%). Performance improvement was statistically significant (p<0.001). Before the sessions, students had the lowest familiarity with Extended Focused Assessment with Sonography in Trauma (eFAST), peripheral nerves, and soft-tissue ultrasound. After the sessions, the majority of students reported being more confident in performing almost all the reviewed applications. The vast majority (90.4%) stated that if possible, they would prefer in-person hands-on sessions for POCUS training.

Conclusion

Medical students’ POCUS knowledge improved significantly after a didactics session and a gamified point-of-care ultrasound distance-learning session. Although the sessions increased their confidence in image acquisition and interpretation, students preferred in-person, hands-on training and practice when given the opportunity.

## Introduction

Ultrasound has been demonstrated as an effective tool in patient care to rapidly evaluate and provide timely intervention with significant utility in various specialties in medicine, such as obstetrics and gynecology, radiology, and emergency medicine. Moreover, focused bedside ultrasonography, in which assessment and management can be delivered by a healthcare provider with a portable ultrasound device, allows for improved patient satisfaction and overall cost-effectiveness [1]. Point-of-care ultrasound (POCUS) training is integrated into graduate medical education, typically through skills training sessions that involve direct interaction between learner and teacher with either simulated models or live models. Studies indicate that there is a greater need for incorporating more clinical experiences, particularly in the initial years of medical education, and ultrasound training would augment these experiences to better prepare medical students for future clinical practice [1,2].

The COVID-19 pandemic proved to be a health crisis with consequential shifts and disruptions in medical education, where many institutions transitioned to alternative online methods of schooling. Some medical schools continued to use online formats for various educational activities such as online lectures, virtual group discussions, and webcasting. While the common trend of medical students using online outside learning resources to supplement recorded school lectures may not have greatly shifted with this transition to institutional online education, some have argued that it is difficult to replicate the invaluable effect of real-time, in-person collaborative education where immediate feedback may be provided to the learner [3]. Skills such as ultrasound techniques that are traditionally taught in live settings are not easily adapted to distance learning. Therefore, there is a need for POCUS educators to become creative in increasing learner interactivity, ideally providing sufficient engagement for comprehension and the acquisition of new skills that would otherwise be carried out in person.

Studies have shown that the “gamification approach,” which involves the use of game design elements in teaching, is a promising method to engage and motivate learners with up to 40% improvement in the ability to learn new skills [4,5]. Given the current state of medical education, the gamification approach may be utilized and adapted to the new transition of online medical education. The successful use of gamification has been demonstrated in ultrasound teaching. Studies have shown that SonoGames, a competitive games-based annual event created in 2011 to educate emergency medicine (EM) residents in POCUS, has been supported as an effective educational tool. Much of its success is attributed to its creation of a competitive and engaging environment where EM residents can participate in challenges that foster teamwork and enthusiasm for ultrasound. A total of 81% of participants in this study reported that their ultrasound knowledge increased, and 87% reported that their enthusiasm for ultrasound increased. Additionally, 61% of participants reported that their clinical use of ultrasound increased after this event [6].

There continues to be limited literature on the effectiveness of gamification in a distance-learning setting when teaching medical students. Therefore, this study aimed to determine whether distance-learning of ultrasound can be performed effectively through gamification techniques and to evaluate the effectiveness and learners perceptions of gamification-based distance learning for POCUS.

This study was previously presented as an oral presentation at the American Institute of Ultrasound in Medicine (AIUM) in 2021.

## Materials and methods

This was a cross-sectional study at an academic center completed in August 2020. Study participants were third-year medical students (MS3) with minimal ultrasound experience. The entire study was conducted through online distance learning. Each participant had access to a computer, a stable internet connection, and Zoom (San Jose, CA: Zoom Communications, Inc.), an online video-conferencing platform. All tests and surveys were administered through Qualtrics (Provo, UT: Qualtrics), an online survey software. Institutional review board approval was obtained for the study.

POCUS fellowship-trained EM faculty developed a 16-question pre-test that assessed students’ prior knowledge of common ultrasonographic findings across multiple systems that were appropriate to their training level. The pre-test was administered to all participants before the didactic session. After the pre-test, POCUS fellowship-trained EM faculty delivered a 1-h traditional online review lecture on basic POCUS applications, including Extended Focused Assessment with Sonography in Trauma (eFAST), cardiac, peripheral nerve, biliary, ultrasound-guided IV placement, soft tissue, obstetrics and gynecology (OB/GYN), and renal imaging.

After the review lecture, students participated in a 1-h gamified online learning session based on the board game Pictionary. During each session turn, a student was randomly selected to draw a POCUS-related structure or concept at the instructor’s discretion. The rest of the students, blinded to the chosen structure, were asked to guess what the selected student was drawing. If students could guess which structure was drawn, they would be awarded a point. After each turn, the instructor would review techniques for image acquisition and teaching points related to the POCUS structure or concept. Multiple turns were taken, and this process was repeated for a variety of concepts that were covered in the initial review lecture.

After the gamified session, students completed a post-test that was identical to the pre-test to assess knowledge retention from the gamified learning session. Students also completed an electronic survey. The survey assessed participants' perceptions of POCUS gamified learning and students' familiarity with POCUS applications before and after the session. A two-sample t-test was performed to determine the statistical significance of the difference between pre-test and post-test scores. For the purposes of statistical analysis, responses on the Likert scale were divided into the following two categories: (1) agreement ("strongly agree" or "agree") and (2) disagreement ("neither agree nor disagree," "disagree," or "strongly disagree"). Survey agreement or disagreement was reported as percentages with 95% confidence intervals. The electronic survey results were reported as Likert scale values (Table 1).

**Table 1:** Student attitude and confidence Likert scale survey.

Survey statements	Strongly disagree	Disagree	Neither agree nor disagree	Agree	Strongly agree
1. Competition-style distance learning is more effective than traditional didactics.	1	2	3	4	5
2. Ultrasound Pictionary via distance learning was an effective method to teach me ultrasound content.	1	2	3	4	5
3. Even though there was no hands-on component this week, the sonographic techniques shown to me increased my confidence to be able to acquire ultrasound views during my rotations.	1	2	3	4	5
4. Learning ultrasound probe manipulation through distance learning is just as effective as in-person hands-on teaching.	1	2	3	4	5
5. If given the opportunity, I would prefer in-person, hands-on ultrasound training rather than distance-learning sessions to learn the various ultrasound applications.	1	2	3	4	5
6. The Ultrasound Pictionary kept me engaged during the session and improved knowledge retention.	1	2	3	4	5

## Results

A total of 73 students completed the pre-test, post-test, and post-session survey. The average pre-test score was 8.53 (53.3%)±2.54 (15.9%), and the average post-test score was 13.44 (84.0%)±2.11 (13.2%). The improvement in performance was statistically significant (p<0.001). Before the ultrasound gamified sessions, students had the lowest familiarity with eFAST, peripheral nerves, and soft-tissue ultrasound. After the sessions, the majority of students reported being more confident in performing all the reviewed applications, except for peripheral nerve ultrasound.

**Figure 1:**
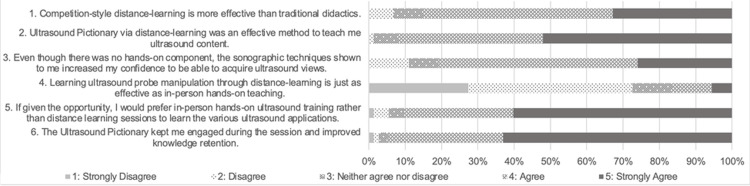
Participant perception of POCUS gamified distance learning. POCUS: point-of-care ultrasound

The survey results of participant perceptions of POCUS gamified distance learning are outlined in Figure 1. A total of 62 students (85%, 95% CI: 81.4-88.4%) thought that competition-style gamified distance learning was more effective than traditional didactics, 67 students (92%, 95% CI: 89.9-93.7%) thought POCUS Pictionary on the video-conferencing platform was an effective method to teach ultrasound content, and 59 students (81%, 95% CI: 69.9-89.1%) stated that even without a hands-on component, the sonographic techniques taught during the gamified session increased their confidence to acquire ultrasound images during their clinical rotations. A total of 69 students (95%, 95% CI: 86.6-98.5%) stated that the POCUS Pictionary session kept them engaged during the session and improved knowledge retention. A total of 53 students (73%, 95% CI: 63.9-84.7%) stated that learning ultrasound probe manipulation via distance learning was just as effective as in-person hands-on teaching. Despite these results, 66 students (91%, 95% CI: 88.2-92.6%) stated that if given the opportunity, they would prefer in-person, hands-on sessions for POCUS training.

## Discussion

Ultrasound education is traditionally taught in person because it is primarily a hands-on modality. The COVID-19 pandemic posed a challenge to ultrasound education due to its hands-on nature. Stay-at-home restrictions were placed on educators and learners, forcing them to adapt quickly. During this time, ultrasound education transitioned to a hybrid model of in-person instruction and video conferencing to accommodate stay-at-home restrictions. Even as these restrictions are lifted, educators have discovered the benefits of this hybrid model, which may continue to be effective post-pandemic [7].

The didactics portion of ultrasound education could certainly be accomplished through distance learning. However, studies have shown that distance learning may make it more difficult to engage learners compared to in-person learning [7]. To combat this, we used gamification, a technique that has been successful in educating residents [7,8]. Our study showed that a majority of students thought competition-style gamification distance learning was more effective than traditional didactics, and the POCUS Pictionary session kept them engaged and improved knowledge retention. Gamification transforms what may have been an unengaging lecture into a fun game, even while students learn from a distance [7].

Our project shows that students can successfully learn at a distance via Pictionary gamification, and this approach resulted in a significant improvement in post-test scores compared to pre-test scores. However, other gamification methods have also been successfully implemented, including Jeopardy, escape room, and treasure hunt [8].

Our study is the first to explore distance learning gamification in ultrasound education for medical students. Previously, SonoGames has shown that gamification is successful in resident ultrasound education [6,9,10]. The pandemic rallied the ultrasound medical education community to come together to provide high-quality resources and ideas that actively engaged learners at all stages. These gamification techniques have been shown to be effective for emergency medicine residents, and we now show that they are effective in medical student education as well.

The students in our study found clinical utility in the application of the ultrasound techniques taught in the gamified distance-learning session, even without a hands-on component. The increase in confidence in probe maneuvering and image acquisition is very promising. This confidence can potentially translate into hands-on skills during in-person instruction and skills practice. This shows that clinically essential and applicable ultrasound skills can still be effectively taught through gamification.

Lastly, while our study successfully utilized distance-learning gamification of ultrasound, a majority of our students would prefer in-person hands-on sessions over distance learning for POCUS training. Similarly, Weber and Ahn showed that residents felt less engaged during distance learning in comparison to in-person learning, but techniques such as gamification improved their distance-learning experience [7].

Our study has several limitations. While this study demonstrated the successful implementation of gamification in ultrasound education for medical students, it was limited to one medical school with a class size of 73 students. Future studies may explore the results of gamification in larger class sizes across various stages of medical education. Our study also relied on students having laptops with cameras and video-conferencing capabilities. These technology requirements may pose a restraint to using distance-learning gamification techniques for students without access to the proper technology. Combating these restraints may require obtaining technological resources from public institutions such as libraries and schools. Additionally, our study taught students using two didactic sessions, but only one of the two sessions was gamified. Our first didactic session created a foundation for students’ ultrasound knowledge base, while the second gamified didactic session likely reinforced the material taught in the first session. Additional studies may be necessary to explore whether the gamification technique can stand alone without a preceding didactic lecture. Future studies may be necessary to explore whether the distance-learning gamification technique can be successfully used to teach hands-on ultrasound skills.

## Conclusions

This study found that students have a positive perception of gamification in POCUS learning topics and that it has a positive effect on content learning. This should be considered when developing distance-learning curricula for POCUS topics. Its overall effectiveness compared to in-person learning warrants further investigation, and further avenues of research could explore other topics in medical education.
